# Characterization of Durum–Einkorn Amphiploids for Introgression of Powdery Mildew Resistance from Einkorn into Common Wheat

**DOI:** 10.3390/pathogens15060653

**Published:** 2026-06-22

**Authors:** Wenting Sheng, Linfeng Chen, Junyu Ma, Muhammad Saqlain, Muhammad Hammad Latif, Ke Zhang, Jingyuan Yang, Muhammad Nosherwan, Wei Zhu, Lili Xu, Dandan Wu, Yonghong Zhou, Chaojie Xie, Houyang Kang, Tzion Fahima, Yinghui Li

**Affiliations:** 1 Triticeae Research Institute, Sichuan Agricultural University, Chengdu 611130, China; shengwenting1018@163.com (W.S.); 19828375120@163.com (L.C.); 15964155093@163.com (J.M.); 2024512001@stu.sicau.edu.cn (M.S.); hammadlatif281@gmail.com (M.H.L.); m15538341372@163.com (K.Z.); y13233088533@163.com (J.Y.); nosherwan320@gmail.com (M.N.); zhuwei202209@163.com (W.Z.); xulili_0627@126.com (L.X.); wudandan@sicau.edu.cn (D.W.); zhouyh@sicau.edu.cn (Y.Z.); houyang.kang@sicau.edu.cn (H.K.); 2 State Key Laboratory of Crop Gene Exploration and Utilization in Southwest China, Sichuan Agricultural University, Chengdu 611130, China; 3 State Key Laboratory of High-Efficiency Production of Wheat-Maize Double Cropping/Frontiers Science Center for Molecular Design Breeding/Key Laboratory of Crop Heterosis and Utilization (MOE)/Beijing Key Laboratory of Crop Genetic Improvement, China Agricultural University, Beijing 100193, China; xiecj127@126.com; 4 Department of Evolutionary and Environmental Biology, Institute of Evolution, University of Haifa, Mt. Carmel, Haifa 3498838, Israel

**Keywords:** amphiploids, common wheat, einkorn wheat, introgression breeding, molecular characterization, powdery mildew

## Abstract

The einkorn wheat group, comprising ancient diploid species (2n = 14, AA), including *Triticum monococcum*, *Triticum boeoticum*, and *Triticum urartu*, represents a valuable source of genetic variation for improving disease resistance in wheat. To develop a practical platform for introgressing powdery mildew resistance into bread wheat, we screened 21 einkorn accessions with *Blumeria graminis* f. sp. *tritici* (*Bgt*) race E09 and identified seven resistant donors. Because direct hybridization between diploid einkorn (AA) and hexaploid wheat (AABBDD) is constrained by genomic divergence and poor cross-compatibility, we crossed resistant einkorn accessions with susceptible durum wheat and induced chromosome doubling in the F_1_ hybrids to generate synthetic durum–einkorn amphiploids. Nine amphiploids were obtained. Chromosome counts and genomic in situ hybridization confirmed the expected genomic constitution (AABBAA) in most lines, with limited variation in chromosome number in two amphiploids. Phenotyping against *Bgt* race E09 showed that three amphiploids retained high resistance, four showed moderate resistance, and two were moderately susceptible. Marker analysis identified five einkorn accessions contain known *Pm* genes such as *Pm60*, *Pm60b*, and *PmNCA6*/*Pm37*, as well as their derived amphipliods. Two einkorn accessions and their derived amphiploids may harbor novel *Pm* genes. Field evaluation of the agronomic traits of these amphiploids indicated some improvement in tillering, spike length, and seed weight. Moreover, these amphiploids had better seed-setting rates in crosses and backcrosses with common wheat. These synthetic durum–einkorn amphiploids thus offer a new bridging platform for transferring alien genes from diploid einkorn to hexaploid common wheat, providing valuable genetic resources for wheat-breeding programs.

## 1. Introduction

Powdery mildew, caused by *Blumeria graminis* f. sp. *tritici* (*Bgt*), is one of the most destructive diseases affecting wheat globally, causing yield losses ranging from 5% to 62% in different countries [1]. The identification, deployment, and utilization of resistance (R) genes are among the most economical and sustainable approaches for controlling wheat powdery mildew. Over 100 powdery mildew resistance genes or chromosomal loci in wheat have been reported, with 71 formally named *Pm* genes (*Pm1* to *Pm71*) [2]. Approximately half of these genes are derived from wild relatives, including *Pm60* from *T. urartu* [3], *Pm68* from *Triticum durum* [4], *Pm69* [5], *MlIW39*/*PmWR183* [6], *Pm41* [7], *Pm26* [8], and *Pm68* from *T. dicoccoides* [4], *Pm21* and *Pm55* from *Dasypyrum villosum* [9,10,11]. These genes, derived from wild relatives, provide an important resource for breeding wheat for disease resistance. However, due to the co-evolution between host resistance and *Bgt* virulence factors (e.g., effectors), some *Pm* genes (e.g., *Pm3*/*Pm8*, *Pm2*, and *Pm6*) have lost their effectiveness [12]. Therefore, identifying new genetic resources for disease-resistant breeding is an ongoing task. Despite the presence of known *Pm* genes in wild wheat, their deployment into cultivated varieties has notably lagged [12]. Accordingly, more focused work is needed to effectively mobilize both known and unknown *Pm* resistance genes from wild germplasm into adapted bread wheat backgrounds.

The einkorn wheat group is among the earliest domesticated wheat species, comprising three main subspecies: wild einkorn wheat, *Triticum boeoticum* Boiss. (2*n* = 2*x* = 14, A^b^A^b^) and *Triticum urartu* Thum. ex Gandil. (2*n* = 2*x* = 14, A^u^A^u^), and cultivated einkorn wheat *Triticum monococcum* L. subsp. *monococcum* (2*n* = 2*x* = 14, A^m^A^m^) [13]. Cultivated einkorn wheat *T. monococcum*, domesticated from its wild progenitor *T. boeoticum*, is the first wheat species known to have been domesticated by humans around 10,000 years ago in the Fertile Crescent [14]. Another close relative is *T. urartu*, the A-genome donor of tetraploid durum wheat (*T. durum*) and hexaploid bread wheat (*T. aestivum*) [15]. This species harbors greater diversity in the A-genome and serves as a valuable source of genetic variation for wheat breeding. For example, several disease resistance genes have been cloned from the einkorn wheat group, including powdery mildew resistance genes *Pm4d*, *Pm37*/*PmNCA6*, and *Pm60* [3,16,17,18], stem rust resistance genes *Sr21*, *Sr22*, *Sr35*, and *Sr60* [19,20,21,22]. *Pm60* was the first powdery mildew resistance gene cloned from *T. urartu* and has three haplotypes: *Pm60*, *Pm60a*, and *Pm60b* [3]; however, it has not been deployed in the commercial common wheat varieties.

Owing to the sterility of hybrids between wild diploid species and common wheat, the “durum as a bridge” strategy can be employed to introgress elite genes into common wheat [23]. Artificially synthesized wheat amphiploids are genetically stable allopolyploid materials, developed through interspecific hybridization between species with different genomes followed by chromosome doubling to restore fertility [24]. Using this method, many amphidiploids and partial amphidiploids have been developed, such as hexaploid triticale (AABBRR), hexaploid tritipyrum (AABBEE), and artificially synthesized hexaploid wheat (AABBDD) [25,26,27]. This process mimics the natural allopolyploidization of common wheat and serves as an effective method for introgressing desirable traits from wild relatives into cultivated wheat. For example, Megyeri et al. [28] developed two allopolyploid combinations using two hybridization approaches (durum wheat/einkorn wheat and emmer wheat/einkorn wheat), both of which showed strong resistance to wheat leaf rust and powdery mildew. Therefore, these amphiploids provide valuable genetic resources and bridging platform for transferring alien genes.

Einkorn wheat harbors valuable *R* gene resources. However, the effective introgression and utilization of these genes in common wheat breeding continue to face major challenges, including reproductive barriers, linkage drag, and genetic instability [12]. This study aims to (i) screen einkorn wheat accessions for resistance to the highly virulent *Bgt* race E09; (ii) develop genetically stable AABBAA synthetic amphidiploids via distant hybridization between durum and einkorn wheat followed by chromosome doubling; (iii) systematically evaluate these amphidiploids for powdery mildew resistance, agronomic traits, and cytogenetic stability; and (iv) use functional markers of known *Pm* genes to determine the presence of known or novel genes. This study provides wheat materials and a theoretical and technical foundation for utilizing einkorn wheat resistance resources in wheat breeding.

## 2. Materials and Methods

### 2.1. Plant Materials

A total of 21 einkorn wheat accessions comprised five accessions of *T. urartu*, eight wild einkorn (*T. boeoticum*), and eight cultivated einkorn (*T. monococcum*) were used in this study for screening powdery mildew resistant lines (Table 1). Two durum wheat lines, Langdon (LDN) and Mo75, were used as tetraploid parents crossing with einkorn wheat to develop amphiploids. Common wheat cultivars Xuezao, Fielder, Mingxian 169 (MX169), Shumai 830 (SM830), and Chuanmai 42 (CM42) were used as parents for distant hybridization. The einkorn wheat accessions were obtained from the Plant Germplasm Resources Institute at the University of Tokyo, Japan, and the information of their origins is available from the Genesys database (accessed on 20 March 2026, https://www.genesys-pgr.org/). Other durum and common wheat lines were obtained from the Triticeae Research Institute, Sichuan Agricultural University.

### 2.2. Development of Synthetic Durum Wheat-Einkorn Wheat Amphiploids

LDN and Mo75 were used as female parents and crossed with einkorn wheat accessions. At the tillering stage, roots of F_1_ hybrid seedlings were washed, older roots were trimmed, and the crown tissue was kept intact. The basal portion of the seedlings was then immersed in 0.1% colchicine solution (optionally supplemented with 1–2% DMSO) in the dark for 4–5 h [29]. Treated seedlings were rinsed under running water for 24 h and transplanted into nutrient soil. The growth conditions were set at 3–15 °C, 50% relative humidity, and a 16 h light/8 h dark photoperiod. The resulting lines displayed high fertility, providing evidence of successful chromosome doubling and their ability to produce viable seeds.

### 2.3. Evaluation of Powdery Mildew Resistance at the Seedling Stage

Healthy wheat seeds were selected, sown in seedling trays, and placed in a growth chamber. The growth conditions were set at 18–21 °C, 50% relative humidity, and a 16 h light/8 h dark photoperiod. The susceptible cultivar Fielder was used as a control. When the first leaf had fully expanded, plants were inoculated with the *Bgt* isolate E09 using the dusting method [30]. Disease reactions were evaluated at 7–10 days after inoculation. When Fielder showed typical disease symptoms, the inoculation was considered successful, and the powdery mildew resistance of the tested wheat lines was recorded. The resistance response was classified into six infection types (ITs) based on the severity of leaf infection: Immune (IT = 0), Immune with cell death (IT = 0;), Highly resistant (IT = 1), Moderately resistant (IT = 2), Moderately susceptible (IT = 3), and Highly susceptible (IT = 4). IT scores of 0–2 were classified as resistant, whereas IT scores of 3–4 were classified as susceptible.

### 2.4. Evaluation of Powdery Mildew Resistance at the Adult Stage

The synthetic amphidiploids, along with their parental lines and susceptible controls, were planted in the field at Wenjiang Huihe Village Experimental Station of Sichuan Agricultural University. The natural disease pressure was provided by susceptible spreader rows that had been inoculated with *Bgt* E09 at the seedling stage. Disease assessments were performed multiple times from heading to grain filling stages (approximately late March) [31], and disease responses were recorded using the same procedure described above.

### 2.5. Cytogenetic Analysis

Chromosome preparation of root-tip mitotic metaphase cells and genomic in situ hybridization (GISH) were performed as described previously [32,33]. Genomic DNA extracted from einkorn wheat was labeled with dUTP-ATO-488 (Jena Bioscience, Jena, Germany) and used as the GISH probe. Total genomic DNA from LDN was used as blocking DNA at a mass ratio of 1:150 (probe:blocking). Chromosomes were counterstained with 4′,6-diamidino-2-phenylindole (DAPI). Images were acquired using an Olympus BX63 fluorescence microscope (Olympus, Tokyo, Japan) equipped with a DP-80 camera.

### 2.6. Detection of Known Resistance Genes

Genomic DNA was extracted from the durum wheat-einkorn wheat amphiploids using the CTAB method [34]. Subsequently, known linked markers or functional markers associated with resistance genes from wheat wild relatives were used to determine whether the resistant materials carried the target resistance genes. The primer sequences of each marker are listed in Table S1. PCR amplification was performed in a total volume of 10 µL, containing 1 µL of template DNA (200 ng/µL), 5 µL of 2 × Taq Master Mix (containing dye, Thermo Fisher Scientific, Bremen, Germany), 1 µL each of forward and reverse primers (10 µM), and 2 µL of ddH_2_O.

The PCR program consisted of an initial denaturation at 95 °C for 5 min, followed by 35 cycles of 95 °C for 15 s (denaturation), 55 °C for 15 s (annealing), and 72 °C for 1 min (extension), with a final extension at 72 °C for 10 min. PCR products were analyzed by electrophoresis on a 1% agarose gel [35].

### 2.7. Agronomic Trait Evaluation

During the 2024–2025 growing season, a field experiment was conducted at the Wenjiang Huihe Village Experimental Station of Sichuan Agricultural University, Chengdu, China, to evaluate the morphological traits of allopolyploid lines. For each line, 15 seeds were sown in 1.5 m rows with 0.3 m spacing. At maturity, 4–5 plants per line were randomly selected and evaluated for morphological traits, including plant height, tiller number, spike length, number of spikelets per spike, number of kernels per spike, and thousand-kernel weight. Significant differences in all measured traits between the allopolyploid lines and their wheat parental lines were analyzed using IBM SPSS Statistics 24.0 software.

### 2.8. Data Analysis

Statistical analyses were performed using IBM SPSS Statistics 24.0. All data are presented as mean ± standard deviation (SD). Comparisons between the synthetic amphidiploids and their respective wheat parental lines for each agronomic trait (plant height, tiller number, spike length, number of spikelets per spike, number of kernels per spike, and thousand-kernel weight) were conducted using one-way analysis of variance (one-way ANOVA). A significance threshold of *p* < 0.05 was considered statistically significant.

## 3. Results

### 3.1. Identification of Resistant Einkorn Accessions to Powdery Mildew

**Figure 1:**
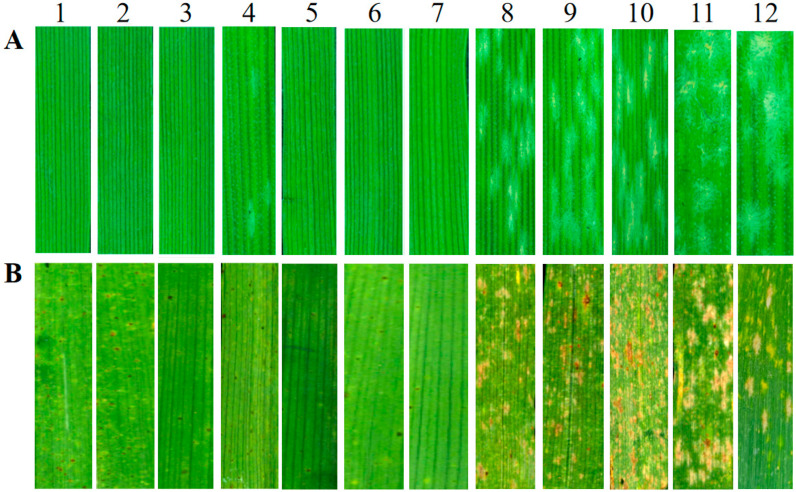
Evaluation of powdery mildew resistance in einkorn wheat and durum wheat accessions. (**A**) Response to *Bgt* E09 at the seeding stage; (**B**) Response to *Bgt* E09 at the adult stage. 1, CITR17664; 2, PI 428215; 3, PI 428315; 4, KU-101-3; 5, KU-104-2; 6, KU-3637; 7, KU-11357; 8, KU-104-3; 9, PI 427500; 10, KU-199-2; 11, Mo75; 12, LDN.

The powdery mildew resistance of 21 einkorn wheat group accessions was evaluated using *Bgt* E09 at the seeding stage (Table 1). The result showed that seven accessions were resistant (IT = 0–2), including three cultivated einkorn wheat (*T. monococcum*) accessions, one wild einkorn wheat (*T. boeoticum*) accession, and three *T. urartu* accessions. Among these, the three *T. urartu* accessions CITR17664, PI 428215, and PI 428315 showed high immunity to *Bgt* E09 (IT = 0); Three *T. monococcum* accessions, KU-104-2, KU-3637, and KU-11357, exhibited high resistance to *Bgt* E09 (IT = 1); and the *T. boeoticum* accession KU-101-3 displayed moderate resistance to *Bgt* E09 (IT = 2) (Figure 1A). However, the durum wheat lines LDN and Mo75 showed high susceptibility to *Bgt* E09 (IT = 4).

In the field conditions, all these einkorn wheat lines showed high resistance (IT = 0), while the durum wheat lines LDN (IT = 3) and Mo75 (IT = 4) showed moderately and highly susceptible in the adult stage (Figure 1B). Therefore, these resistant einkorn accessions were prioritized as donor parents to develop marker-trackable introgression materials.

### 3.2. Development and Cytological Validation of Durum–Einkorn Wheat Amphiploids

**Figure 2:**
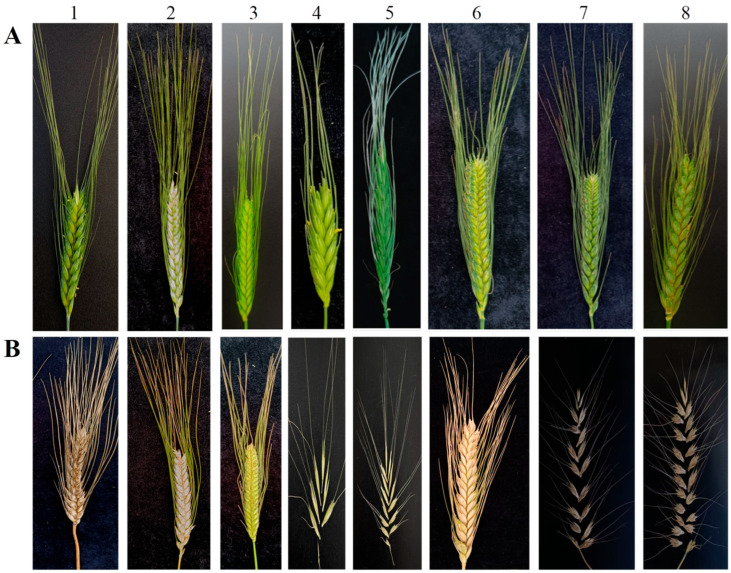
Spike morphology of einkorn wheat, durum wheat, and artificially synthesized amphiploids. (**A**) Young spike stage; (**B**) Mature spike stage. 1, Mo75; 2, LDN; 3, A^m^A^m^(KU-3637); 4, A^b^A^b^(KU-101-3); 5, A^u^A^u^(PI 428315); 6, AABBA^m^A^m^ (Mo75/KU-3637); 7, AABBA^b^A^b^ (LDN/KU-101-3); 8, AABBA^u^A^u^ (Mo75/PI 428315).

In order to transfer the powdery mildew resistance gene from einkorn wheat into cultivated bread wheat, seven disease-resistant einkorn wheat accessions were crossed with susceptible durum wheat accessions LDN and/or Mo75. Chromosome doubling was induced by colchicine treatment at the F_1_ tillering stage, resulting in the synthesis of durum–einkorn wheat amphiploids. Nine synthetic amphiploids were obtained with high fertility and capable of self-fertilization (Table 2). The spike morphology of these amphiploids showed distinct differences with their parental lines with improved biomass size (Figure 2A). Moreover, in some amphiploids, the brittle rachis trait was genetically derived from *T. urartu* and wild einkorn wheat (Figure 2B), confirming the legitimacy of the hybridization event.

**Figure 3:**
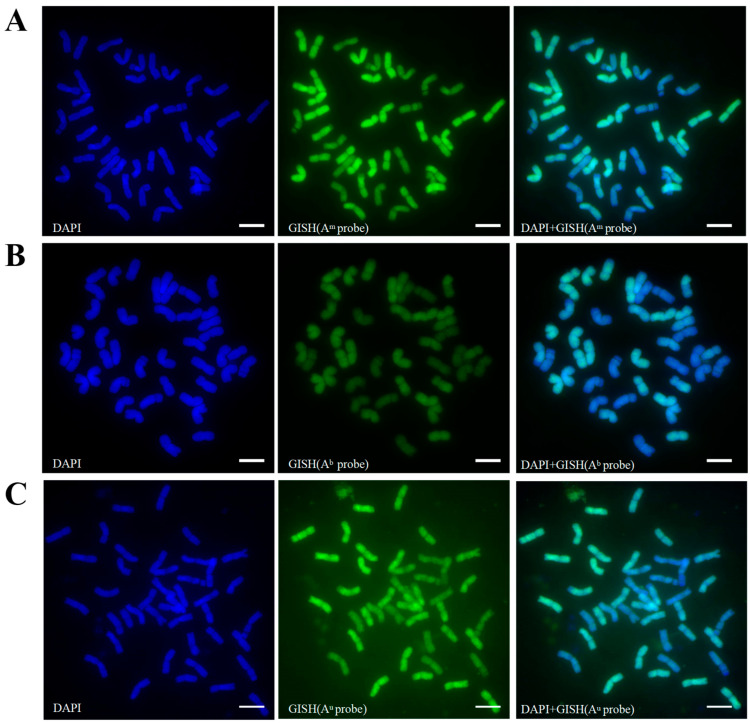
Cytological identification of durum–einkorn wheat amphiploids. *Triticum monococcum* L. (KU-3637) and *Triticum boeoticum* (KU-101-3) genome DNA were labeled in green as GISH probe. (**A**) GISH identification of LDN/KU-3637. Chromosomes were counterstained with DAPI (blue), A and A^m^ genomes were labeled with bright green signals, while the B genome showed weak green signals. (**B**) GISH identification of LDN/KU-101-3. Chromosomes were counterstained with DAPI (blue), A and A^b^ genomes were labeled with bright green signals, while the B genome showed weak green signals. (**C**) GISH identification of Mo75/CITR17664. Chromosomes were counterstained with DAPI (blue), A and A^u^ genomes were labeled with bright green signals, while the B genome showed weak green signals. Scale bars, 10 µm.

Cytological analyses were conducted on the selfed progenies (F_3_ generations) of the synthetic amphiploids. Chromosome counts at mitotic metaphase indicated that seven synthetic amphiploids carried the expected chromosome number of 42, suggesting overall genome stability, whereas LDN/KU-104-2 and Mo75/PI 428315 carried 41 and 43 chromosomes, respectively (Table S2 and Figure S1). We further performed genomic in situ hybridization (GISH) on three synthetic amphiploids to characterize their genomic composition (Figure 3A–C). Total genomic DNA of einkorn wheat was labeled as a green probe. Of the 42 chromosomes in the synthetic amphiploids, 28 chromosomes from the A genome exhibited strong green fluorescent signals, while 14 B-genome chromosomes displayed weak signals. These GISH results, combined with the mitotic chromosome observations, further validated the relatively genomic stability of the synthetic amphiploid materials.

### 3.3. Powdery Mildew Infection Types of Amphiploids at the Seeding and Adult Stages

We phenotyped the synthetic amphiploids to *Bgt* E09 in a growth chamber at the seedling stage (Table 2). The results showed that the durum parental lines Mo75 and LDN were highly susceptible to *Bgt* E09 (IT = 4) (Figure S2A). In contrast, three amphiploids (Mo75/CITR17664, Mo75/PI 428215, and LDN/KU-104-2) were highly resistant to powdery mildew (IT = 0–1), four amphiploids (Mo75/KU-3637, Mo75/KU-11357, LDN/KU-11357, and Mo75/PI 428315) exhibited moderate resistance (IT = 2). The remaining two amphiploids (LDN/KU-3637 and LDN/KU-101-3) were moderately susceptible to *Bgt* E09 (IT = 3) (Figure S2A). Specifically, amphiploids derived from CITR17664, PI 428215, and KU-104-2 maintained high resistance (IT = 0–1), while those from KU-3637, KU-11357, and PI 428315 maintained moderate resistance (IT = 2). However, the amphiploid derived from KU-101-3 showed reduced resistance (IT = 3 at seedling stage) compared to its donor (IT = 2), indicating background-dependent expression (Table 2). These results indicate that resistance derived from einkorn wheat can be retained after amphiploid formation, but its phenotypic expression is background-dependent across donor combinations.

Meanwhile, adult-plant resistance to powdery mildew was also evaluated in the field (Table 2). The results showed that six lines (Mo75/KU-3637, Mo75/KU-11357, LDN/KU-11357, LDN/KU-104-2, Mo75/CITR17664, Mo75/PI 428215, and Mo75/PI 428315) were highly resistant to powdery mildew (IT = 0–1), two lines (LDN/KU-3637 and LDN/KU-101-3) exhibited moderate resistance (IT = 2) (Table 2). However, the durum parent lines LDN showed moderate susceptibility (IT = 3), and Mo75 showed high susceptibility (IT = 4) at the adult stage (Figure S2B). This result suggested that most amphiploids exhibited improved resistance to powdery mildew at the adult stage.

### 3.4. Marker-Defined Resistance Classes in Einkorn Donors and Derived Amphiploids

**Figure 4:**
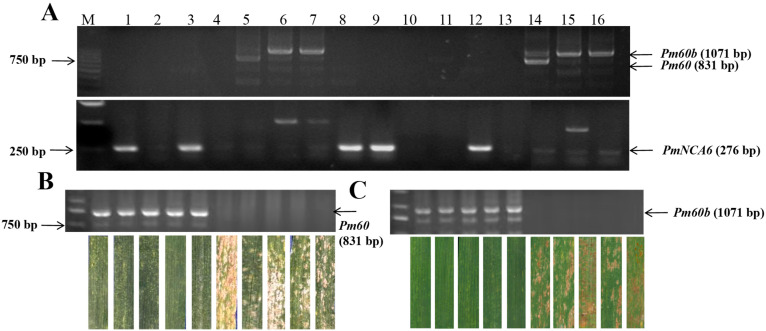
A genome gene detection in durum–einkorn wheat amphidiploids and their parental lines and introgression of *Pm60* and *Pm60b* into common wheat backgrounds. (**A**) The arrows indicate the specific bands. M, D2000; 1, KU-101-3; 2, KU-104-2; 3, KU-3637; 4, KU-11357; 5, CITR17664; 6, PI 428215; 7, PI 428315; 8, Mo75/KU-3637; 9, LDN/KU-3637; 10, Mo75/KU-11357; 11, LDN/KU-104-2; 12, LDN/KU-101-3; 13, LDN/KU-11357; 14, Mo75/CITR17664; 15, Mo75/PI 428215; 16, Mo75/PI 428315. The linkage analysis of *Pm60-M-S1* and *Bgt* resistance, and the PCR products result in the presence of the *Pm60* allele (**B**) and *Pm60b* allele; (**C**) The morphology of introgression lines with *Pm60* allele.

Seven einkorn wheat accessions and nine synthetic amphiploids were screened using functional or closely linked markers for known powdery mildew resistance genes (*Pm1a*, *Pm3b*, *Pm4d*, *Pm37*, *PmNCA6*, and *Pm60*), which were located on the A genome. One *T. urartu* accession (CITR17664) carried *Pm60*, and two *T. urartu* accessions (PI 428215 and PI 428315) were found to contain *Pm60b* alleles (Figure 4A). Their corresponding synthetic amphiploid (Mo75/CITR17664, Mo75/PI 428215 and Mo75/PI 428315) also contains these *Pm60* alleles, which were inherited from the resistant *T. urartu* parents. Moreover, one *T. boeoticum* accession (KU-101-3) and one *T. monococcum* accession (KU-3637), and their corresponding synthetic amphiploids (Mo75/KU-3637, LDN/KU-3637 and LDN/KU-101-3), contain *PmNCA6*/*Pm37* (Figure 4A and Figure S3). Conversely, the absence of the *Pm1a*, *Pm3b*, and *Pm4d* genes in other resistant amphiploids (Mo75/KU-11357, LDN/KU-11357, and LDN/KU-104-2) suggests that they may harbor novel powdery mildew resistance genes (Figure S3).

### 3.5. Transmission of Resistance into Bread Wheat Backgrounds

To test whether amphiploid-derived resistance could be transmitted into bread wheat, four resistant amphiploids (Mo75/KU-3637, Mo75/KU-11357, LDN/KU-11357, and LDN/KU-104-2) were crossed with the susceptible cultivar MX169. The resulting F_1_ plants showed moderate resistance (IT = 2) to *Bgt* E09 at the seedling stage (Figure S4A), while showing high resistance (IT = 0) at the adult stage (Figure S4B). This result suggested that the resistance genes from amphiploids could be used to improve *Bgt* resistance in common wheat. Notably, F_1_ hybrids exhibited longer spikes and more spikelet numbers per spike (n = 30) than MX169 (n = 20), suggesting the great potential for breeding (Figure S5). However, the F_1_ hybrids showed sterility, with extremely low self-fertility (~5%) (Figure S5). To restore fertility and facilitate gene introgression, the F_1_ plants were backcrossed as female parents with CM42, Fielder, and SM830, and their fertility gradually increased (~20%). This finding lays the foundation for developing pre-breeding lines that combine powdery mildew resistance from einkorn wheat with desirable agronomic traits.

Using these methods, we previously transferred the *Pm60* and *Pm60b* alleles from *T. urartu* into susceptible wheat cultivars, obtaining two retrogression lines [36]. We crossed the introgression lines with susceptible common wheat cultivar CM42, which yielded two F_2_ populations containing *Pm60* (Mo75/CITR 17664//Xuezao//Fielder///CM42) and *Pm60b* (Mo75/PI 428215//Xuezao///Fielder///CM42), respectively. Molecular marker analysis of the F_2_ population demonstrated that the *Pm60* and *Pm60b* alleles are associated with *Bgt* resistance (Figure 4B,C). These results confirm the successful introgression of both alleles into the common wheat genetic background, where they confer resistance to powdery mildew. Importantly, both *Pm60* and *Pm60b* showed no apparent linkage drag, positioning them as excellent pre-breeding lines for improving wheat resistance.

### 3.6. Evaluation of Agronomic Traits in Amphiploids

**Figure 5:**
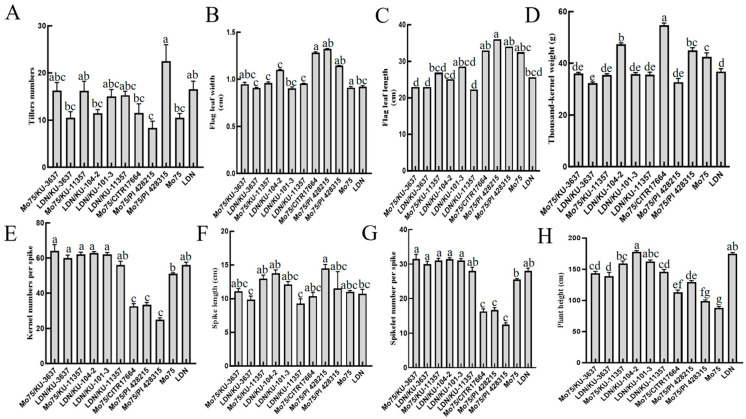
Agronomic traits in artificially synthesized amphidiploids and their parental lines. (**A**) Tiller numbers; (**B**) Flag leaf width (cm); (**C**) Flag leaf length (cm); (**D**) Thousand-kernel weight (g); (**E**) Kernel number per spike; (**F**) Spike length (cm); (**G**) Spikelet number per spike; (**H**) Plant height (cm). Groups that do not share a common letter (a–g) are significantly different.

During the 2024–2025 wheat growing season, the agronomic traits of amphiploids and their parental lines were evaluated at the maturity stage. Compared with the parental durum lines, these amphiploids showed significant differences in many agronomic traits, such as more tillering numbers (Mo75/KU-3637, Mo75/KU-11357, and Mo75/PI 428315) (Figure S5A–C and Figure 5, Table S3), increase in grain length, grain width, and grain size (Mo75/CITR 17664 and Mo75/PI 428215) (Figure S5D), increased thousand-kernel weight (Mo75/CITR 17664 and LDN/KU-104-2) and more kernel per spike (Mo75/KU-3637, Mo75/KU-11357, LDN/KU-104-2, and LDN/KU-101-3) (Figure 5, Table S3). These trait differences indicate that several amphiploids combine resistance with agronomically useful characteristics, although additional backcrossing will be needed to reduce undesirable domestication-related traits, such as brittle rachis and prostrate habit.

## 4. Discussion

### 4.1. The Einkorn Wheat Group Is a Genetic Resource in Wheat Resistance Breeding

The einkorn wheat group represents a valuable genetic resource for improving disease resistance in cultivated wheat. Several important resistance genes have been derived from einkorn wheat and successfully transferred to common wheat. For stem rust resistance, the Ug99-resistance genes *Sr21*, *Sr22b*, *Sr35*, *SrTm4*, and *Sr60* originated from *T. monococcum* [19,20,22,37,38,39]. For powdery mildew resistance, three functional alleles of *Pm60* (*Pm60*, *Pm60a*, and *Pm60b*) were cloned from *T. urartu* [3]. *Pm37*, a susceptible allele of *Sr22*, confers resistance to powdery mildew and leaf rust in wheat, which is thought to originate from *T. monococcum* and was introduced into common wheat via *Triticum timopheevii* [17]. *PmNCA6*, homologous to *Sr22a*, was cloned from *T. boeoticum* and has been transferred into the advanced breeding line YangYF267, which was not affected by regional *Bgt* isolates and lacks linkage drag [18]. Additionally, *PmTb7A.1*, *PmTb7A.2* and *QYrtb.pau-5A* were identified from wild einkorn wheat [40,41]. *Mlm2033*, *Mlm80*, and *pm2026* were identified from *T. monococcum* [42,43]. Moreover, the stripe rust resistance gene *Yr34*/*Yr48* was identified in *T. monococcum* [44]. Our study identified seven einkorn wheat lines resistant to powdery mildew, and molecular marker analysis showed that five accessions carry known *Pm* genes (e.g., *Pm60*, *Pm60b*, and *Pm37*/*PmNCA6*), and two accessions (KU-104-2 and KU-1135) may carry potentially unknown *Pm* genes (Figure 4A and Figure S2). Overall, these diploid einkorn wheat relatives offer valuable genetic resources for wheat resistance breeding.

### 4.2. Allopolyploid Genetic Stability and Breeding Potential

Cytological analyses (GISH and FISH) revealed that most of the durum–einkorn amphiploids developed in this study exhibited relatively stable chromosome numbers (2n = 42) (Figure 3 and Figure S1). This finding is consistent with Michikawa et al. [45], who also obtained stable hexaploid wheat lines by crossing LDN with wild einkorn, indicating that the AABBAA configuration possesses good meiotic stability. In this study, most amphiploids derived from the seven resistant einkorn donors retained the high-resistance phenotype (IT = 0–2) of their parents (Table 2). After crossing the amphiploids with common wheat, the F_1_ hybrids were partial fertile with only 5% seeds setting probably because of the failure of chromosome pairing. However, the F_1_ plants were backcrossed as female parents with common wheat, and their fertility gradually increased (~20%). This suggested that the amphiploids could be easily used as bridging platform for transferring alien genes from diploid einkorn to hexaploid common wheat. However, it is worth noting that some amphiploids (e.g., LDN/PI 427724) became susceptible (IT = 3), even though their einkorn parent was resistant. This discrepancy may be explained by polyploidy-induced epigenetic silencing and genetic background interactions [46]. In this case, different wheat genetic backgrounds can be used to test the resistance efficiency of these *Pm* genes in future.

The synthetic hexaploid wheat (SHW) as a bridge to transfer resistance genes from *Aegilops tauschii* has been used widely in wheat breeding. For example, the core parent used in the breeding of Chuanmai 42 was Syn-CD768, a SHW developed by CIMMYT (original cross: durum wheat Altar84 × *Aegilops tauschii* 188), using the same method, the SHW-derived cultivars, namely, Chuanmai 38, 43, 47, and 104, have been raised and cultivated [47,48]. It became the first registered wheat cultivar in the world successfully bred from a synthetic hexaploid wheat that could be directly extended for production. Xiaoyan 6 was obtained by crossing common wheat with *Thinopyrum* ponticum, producing a partial amphiploid (AABBDDEE), which was widely planted in the Huang–Huai wheat region. This suggests that the amphiploids developed in this study could serve as valuable genetic resources for wheat-breeding programs, as they are easier to utilize than through direct distant crossing with wild relatives.

### 4.3. Current Status of Einkorn Wheat Utilization in Breeding

As the donor species of the A genome of common wheat, the einkorn wheat group harbors abundant disease-resistance gene resources and has made significant progress in disease-resistance breeding in recent years [44]. For example, Chen et al. [19] cloned *Sr60* (also known as WTK2) from cultivated einkorn wheat. This gene confers resistance to the Ug99 stem rust race and was successfully transferred into hexaploid wheat as a small einkorn fragment without linkage drag. Another example is *Yr34*/*Yr48* from *T. monococcum*, which was translocated to chromosome 5AL in polyploid wheat. The old European wheat variety “Mediterranean” was identified as a putative source of this translocation, suggesting that *Yr34* has been used for over 200 years [44]. This supports that genes from einkorn wheat are not only rich sources of disease resistance but also compatible with polyploid wheat genomes, enabling durable and linkage-drag-free introgression for modern wheat-breeding programs.

In this study, the nine AABBAA amphidiploid materials were developed showed improved agronomic traits and can serve as donors in crosses and backcrosses with high-yielding common wheat cultivars to transfer elite genes into common wheat. The F_1_ generation derived from crosses between AABBAA amphiploids and common wheat (AABBDD) exhibited enhanced powdery mildew resistance, suggesting the disease resistance genes could be functional in the common wheat background (Figure S6). The *Pm60* alleles showed high resistance to *Bgt* in the common wheat background without apparent linkage drag, positioning them as excellent pre-breeding lines for improving wheat resistance (Figure 4). Future research should focus on cloning and functionally characterizing resistance genes, developing small-fragment translocation lines for precise gene transfer, and constructing multi-gene pyramided systems for durable resistance. These efforts will fully realize the strategic value of einkorn wheat in broadening the genetic basis of disease resistance in wheat.

## 5. Conclusions

To facilitate introgression of powdery mildew resistance from diploid einkorn (AA) into hexaploid bread wheat (AABBDD), nine synthetic durum–einkorn amphiploids (AABBAA) were developed via hybridization and chromosome doubling. Three amphiploids retained high resistance to *Bgt* race E09, with five carrying known *Pm* genes (*Pm60*, *Pm60b*, and *PmNCA6*/*Pm37*) and two potentially harboring novel genes. These amphiploids also showed improved agronomic traits and better crossability with common wheat, serving as a valuable bridging platform for wheat-breeding programs. The methods employed in this study offer important guidance for future research (Table S4).

## Figures and Tables

**Table 1:** Information and *Bgt* resistance evaluation of einkorn wheat accessions.

Number	ID	Collection Site	Species	Chromosome	IT
1	KU-104-3	-	*T. monococcum*	A^m^A^m^	4
2	KU-105	-	*T. monococcum*	A^m^A^m^	4
3	KU-1001	-	*T. monococcum*	A^m^A^m^	4
4	KU-1404	-	*T. monococcum*	A^m^A^m^	4
5	KU-11072	Turkey	*T. monococcum*	A^m^A^m^	3
6	KU-3637	Turkey	*T. monococcum*	A^m^A^m^	1
7	KU-11357	Romania	*T. monococcum*	A^m^A^m^	1
8	KU-104-2	-	*T. monococcum*	A^m^A^m^	1
9	PI 427496	Turkey	*T. boeoticum*	A^b^A^b^	3
10	PI 427500	Turkey	*T. boeoticum*	A^b^A^b^	4
11	PI 427666	Iraq	*T. boeoticum*	A^b^A^b^	4
12	PI 427667	Iraq	*T. boeoticum*	A^b^A^b^	3
13	PI 427719	Iraq	*T. boeoticum*	A^b^A^b^	3
14	PI 427838	Iraq	*T. boeoticum*	A^b^A^b^	3
15	PI 427444	Turkey	*T. boeoticum*	A^b^A^b^	4
16	KU-101-3	-	*T. boeoticum*	A^b^A^b^	2
17	KU-199-2	-	*T. urartu*	A^u^A^u^	4
18	KU-199-8	-	*T. urartu*	A^u^A^u^	3
19	CITR17664	Lebanon	*T. urartu*	A^u^A^u^	0
20	PI 428215	Turkey	*T. urartu*	A^u^A^u^	0
21	PI 428315	Lebanon	*T. urartu*	A^u^A^u^	0

Note: *Bgt* race E09 was used for phenotyping; IT, Infection type at seeding stage. Accessions denoted by a dash (“-”) lack collection site information, as these details were not provided in the original germplasm passport records. The superscript letters “m”, “b”, and “u” denote the genomes of *Triticum monococcum*, *Triticum boeoticum*, and *Triticum urartu*, respectively.

**Table 2:** The phenotypic responses of durum–einkorn wheat amphiploids to *Bgt* E09 at both seedling and adult stages.

Hybrid Combination	Chr.	IT-Seeding	IT-Adult
Mo75	AABB	4	4
LDN	AABB	4	3
Mo75/KU-3637	AABBA^m^A^m^	2	0
LDN/KU-3637	AABBA^m^A^m^	3	2
Mo75/KU-11357	AABBA^m^A^m^	2	1
LDN/KU-11357	AABBA^m^A^m^	2	0
LDN/KU-104-2	AABBA^m^A^m^	1	0
LDN/KU-101-3	AABBA^b^A^b^	3	2
Mo75/CITR17664	AABBA^u^A^u^	0	0
Mo75/PI 428215	AABBA^u^A^u^	0	0
Mo75/PI 428315	AABBA^u^A^u^	2	0

Note: IT, Infection type at adult stage; IT = 0–2, resistant; IT = 3–4, susceptible. The superscript letters “m”, “b”, and “u” denote the genomes of *Triticum monococcum*, *Triticum boeoticum*, and *Triticum urartu*, respectively.

## Data Availability

The genotype data and the plant materials reported in this study are available upon request.

## Supplementary Materials

The following supporting information can be downloaded at: https://www.mdpi.com/article/10.3390/pathogens15060653/s1. Figure S1. Observation of mitotic metaphase chromosomes in durum–einkorn amphiploids. Figure S2. Evaluation of powdery mildew disease resistance of durum–einkorn wheat amphiploids and their parental durum wheat lines. Figure S3. Marker detection of known *Pm* genes from wheat A–genomes in durum–einkorn amphiploids and their parental lines. Figure S4. Evaluation of powdery miledew disease resistance in the F_1_ generation of durum–einkorn amphidiploids crossed with common wheat. Figure S5. Spike morphology of the F1 generation of durum–einkorn amphidiploids crossed with common wheat. Figure S6. Agronomic traits in durum–einkorn amphidiploids and their parental lines. Table S1. Marker detection of known *Pm* genes from the wheat A–genome. Table S2. Chromosome counting in durum–einkorn amphiploids. Table S3. Investigation of agronomic traits in synthetic amphiploids. Table S4. Summarizes the main steps for developing and characterizing the synthetic durum-einkorn amphiploids. References [49,50,51] are cited in the Supplementary Materials.
