# Supplementary material for: Characterization of Durum–Einkorn Amphiploids for Introgression of Powdery Mildew Resistance from Einkorn into Common Wheat
Source: Pathogens. 2026 Jun 22;15(6):653. doi: 10.3390/pathogens15060653 (PMC13304744; doi:10.3390/pathogens15060653)
Supplement: Supplementary file 1 [file pathogens-15-00653-s001.zip › pathogens-4340587-supplementary.pdf]

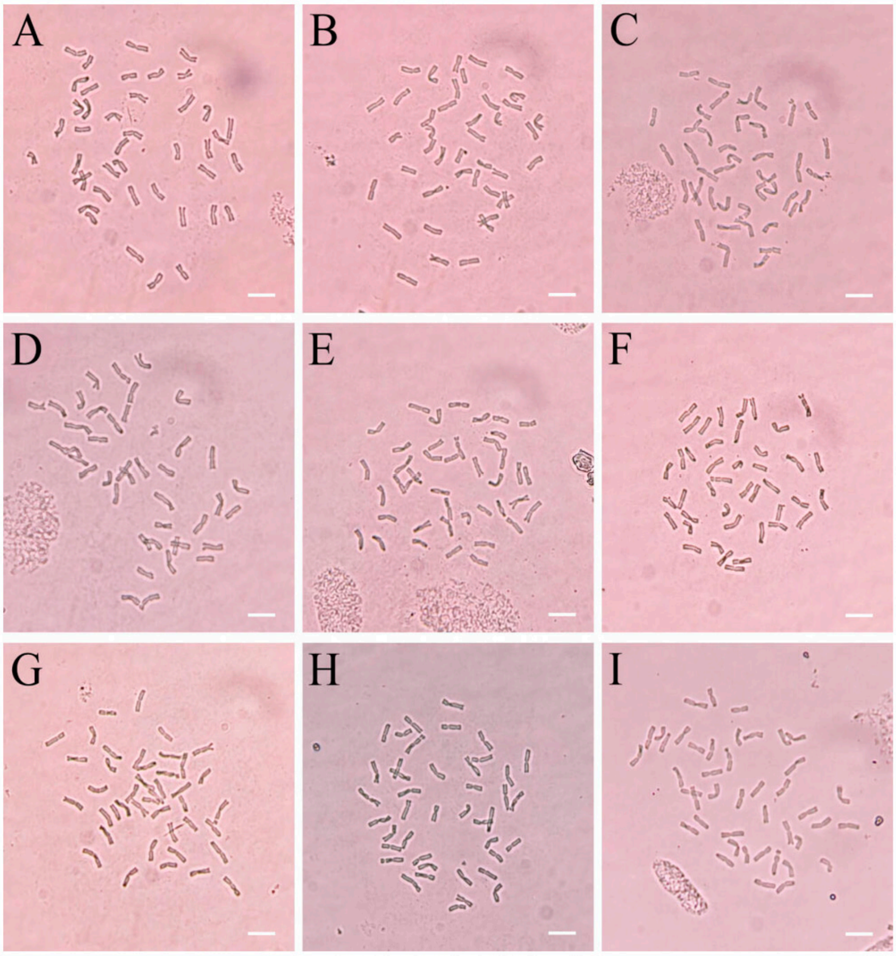

Figure S1. Observation of mitotic metaphase chromosomes in durum–einkorn amphiploids. (**A**) Mo75/KU-3637; (**B**) LDN/KU-3637; (**C**) Mo75/KU-11357; (**D**) LDN/KU-104-2; (**E**) LDN/KU-101-3; (**F**) LDN/KU-11357; (**G**) Mo75/CITR17664; (**H**) Mo75/PI 428215; (**I**) Mo75/PI 428315. Scale bars, 10 μm.

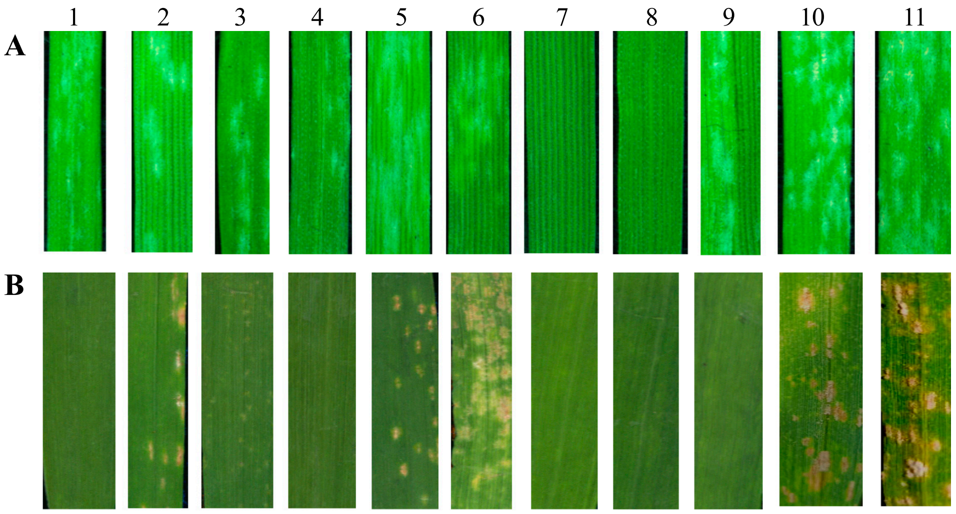

Figure S2. Evaluation of powdery mildew disease resistance of durum–einkorn wheat amphiploids and their parental durum wheat lines. (**A**) Infection types to *Bgt* E09 at seedling stage; (**B**) Infection types to *Bgt* E09 at adult stage. 1, Mo75/KU-3637; 2, LDN/KU-3637; 3, Mo75/KU-11357; 4, LDN/KU-104-2; 5, LDN/KU-101-3; 6, LDN/KU-11357; 7, Mo75/CITR17664; 8, Mo75/PI 428215; 9, Mo75/PI 428315; 10, Mo75; 11, LDN.

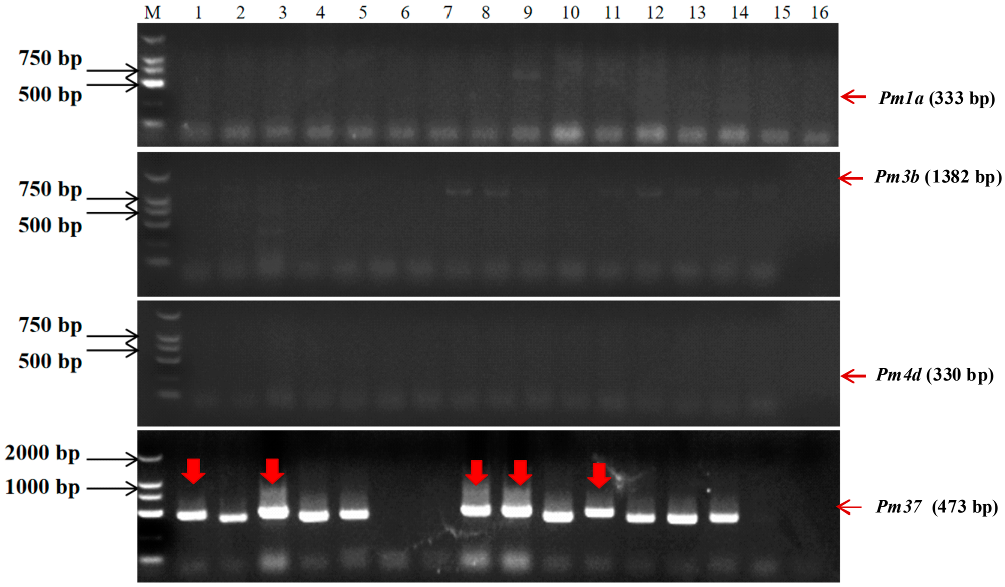

Figure S3. Marker detection of known *Pm* genes from wheat A–genomes in durum–einkorn amphiploids and their parental lines. The red arrows indicate the expected *Pm*-gene specific band locations. M, D2000 DNA marker; 1, KU-101-3; 2, KU-104-2; 3, KU-3637; 4, KU-11357; 5, CITR17664; 6, PI 428215; 7, PI 428315; 8, Mo75/KU-3637; 9, LDN/KU-3637; 10, Mo75/KU-11357; 11, LDN/KU-104-2; 12, LDN/KU-101-3; 13, LDN/KU-11357; 14, Mo75/CITR17664; 15, Mo75/PI 428215; 16, Mo75/PI 428315. The *Pm37* and *PmNCA6* showed same sequence, therefore they showed the same results of marker detection.

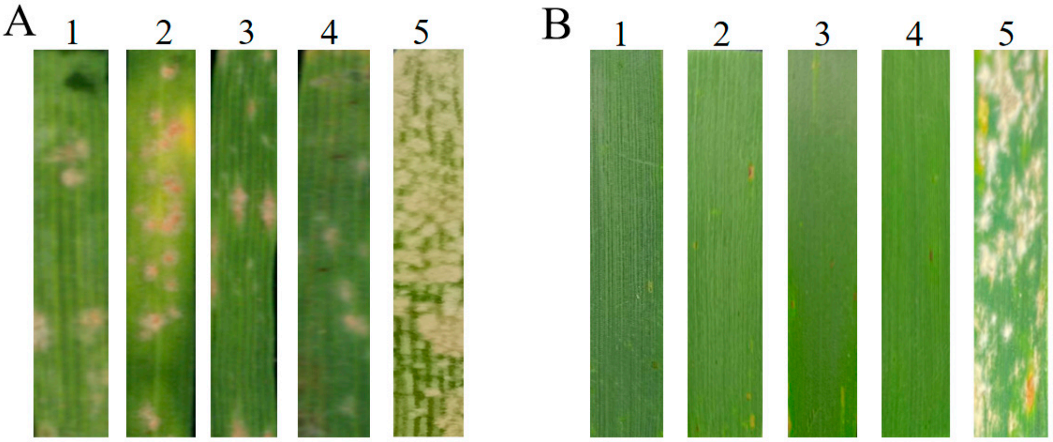

Figure S4. Evaluation of powdery miledew disease resistance in the F_1 generation of durum–einkorn amphidiploids crossed with common wheat. (**A**) Infection types at seedling stage; (**B**) Infection types at adult stage. 1, MX169//Mo75/KU-3637; 2, MX169//Mo75/KU-11357; 3, MX169//LDN/KU-11357; 4, MX169//LDN/KU-104-2; 5, MX169.

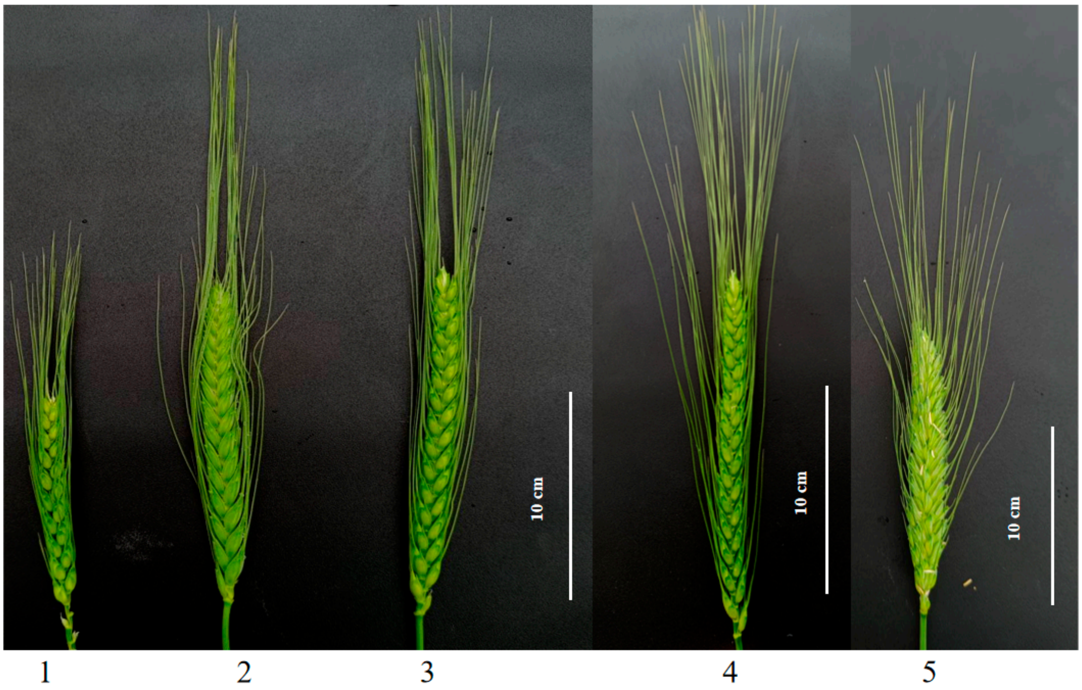

Figure S5. Spike morphology of the F_1 generation of durum-einkorn amphidiploids crossed with common wheat. 1, MX169; 2, Mo75/KU-11357; 3, MX169/Mo75/KU-11357; 4, MX169/Mo75/KU-3637; 5, MX169/Mo75/KU-11357.

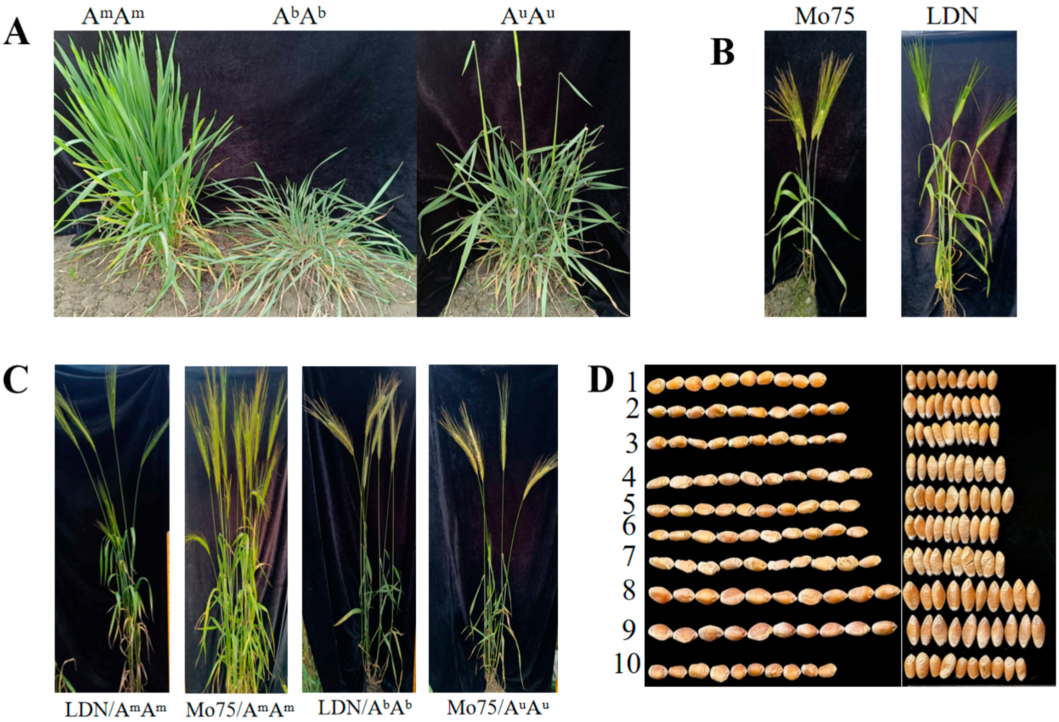

Figure S6. Agronomic traits in durum–einkorn amphidiploids and their parental lines. (**A**) The plant morphology of *Triticum monococcum* (A^mA^m, KU-3637), *Triticum boeoticum* (A^bA^b, KU-101-3), and *Triticum urartu* (A^uA^u, CITR17664). (**B**) The plant morphology of durum wheat Mo75 and LDN (**C**) The plant morphology of durum-einkorn amphidiploids LDN/A^mA^m (KU-3637), Mo75/A^mA^m (KU-11357), LDN/A^bA^b (KU-101-3) and Mo75/A^uA^u (CITR17664); (**D**) The seed morphology of durum–einkorn amphidiploids and the durum accessions; 1, Mo75; 2, Mo75/KU-3637; 3, LDN/KU-3637; 4, Mo75/KU-11357; 5, LDN/KU-104-2; 6, LDN/KU-101-3; 7, LDN/KU-11357; 8, Mo75/CITR 17664; 9, Mo75/PI 428215; 10, Mo75/PI 428315.

Table S1. Marker detection of known *Pm* genes from the wheat A‒genome.

Gene	Marker name	Chr. location	Forward (F) and reverse (R) primer sequences (5' to 3')	Amplicon length	Reference
Pm1a	*Pm1aSTS1*	7A	F:CAATATAAACTTCAGATGTTCTATT CTCAAAA<br>R: CTACATTGGCTATGCGTGTAGTC	333 bp	[49]
Pm3b	*Pm3b/F*	1A	F: GGCACAGACAAAGCTCTG<br>R: TCGAGTAGCTCGGGAATC	1382 bp	[50]
Pm4d	*ResPm4*	2A	F:TGTCCGGTTTGGTTACCTTTCTTC<br>R: GGGACGCTTCCTATAATCACGC	330 bp	[16]
Pm37	*Pm37-FM*	7A	F: TGTCAAACTTGCGGCTAT<br>R: TGCTACGAAACCAGGAAA	473 bp	[17]
PmNCA6	*FM-NLR*	7A	F: TACACGCCAGACTCCCTAAC<br>R: GGACAACACCCTGCCATTG	276 bp	[18]
Pm60	*M-Pm60-S1*	7A	F: CTCACAGTTCCACACTGATAT<br>R: CTCCATCAATCTCAAGTTCTTCG	831 bp	[51]

Table S2. Chromosome counting in durum‒einkorn amphiploids.

Hybrid combination	Chromosome constitution	Chromosome number
Mo75/KU-3637	AABBA^{m}A^{m}	42
LDN/KU-3637	AABBA^{m}A^{m}	42
Mo75/KU-11357	AABBA^{m}A^{m}	42
LDN/KU-11357	AABBA^{m}A^{m}	42
LDN/KU-104-2	AABBA^{m}A^{m}	41
LDN/KU-101-3	AABBA^{b}A^{b}	42
Mo75/CITR17664	AABBA^{u}A^{u}	42
Mo75/PI 428215	AABBA^{u}A^{u}	42
Mo75/PI 428315	AABBA^{u}A^{u}	43

Note: The superscript letters "m", "b", and "u" denote the genomes of *Triticum monococcum*, *Triticum boeoticum*, and *Triticum urartu*, respectively.

Table S3. Investigation of agronomic traits in synthetic amphiploids.

Hybrid combination	Tiller numbers	Plant height (cm)	Spike length (cm)	Flag leaf length (cm)	Flag leaf width (cm)	Spikelet number per spike	Kernel numbers per spike	Thousand -kernel weight (g)
Mo75	10.5±1.7	87.8±5.7	11.0±0.4	32.5±2.9	2.2±0.2	25.5±0.6	51.0±1.2	42.5±3.0
LDN	16.5±3.5	175.0±3.7	10.8±1.3	25.6±4.0	1.6±0.1	28.0±1.6	56.0±3.3	36.8±2.2
Mo75/KU-3637	16.3±3.4	146.9±10.7	11.9±1.1	22.9±3.5	1.8±0.1	32.0±3.7	64.0±7.3	36.0±0.8
LDN/KU-3637	10.5±2.5	138.8±12.7	9.9±1.0	23.0±2.7	1.4±0.2	30.0±1.6	60.0±3.3	32.3±1.0
Mo75/KU-11357	16.2±4.6	159.0±9.6	13.0±1.1	26.9±5.1	1.5±0.1	31.0±1.4	62.0±2.8	35.4±1.1
LDN/KU-104-2	11.4±1.8	178.0±4.3	13.8±1.0	25.1±1.3	1.6±0.2	31.4±0.9	62.8±1.8	47.4±1.1
LDN/KU-101-3	15.0±2.9	162.3±5.3	12.1±0.9	28.6±3.8	1.7±0.1	31.0±1.2	62.0±2.3	35.8±1.3
LDN/KU-11357	15.3±1.7	146.0±7.4	9.3±1.4	22.3±2.6	1.4±0.1	28.0±2.2	56.0±4.3	35.5±2.1
Mo75/CITR17664	11.5±3.9	113.1±7.2	10.4±1.1	33.0±3.6	2.2±0.5	16.3±1.5	32.5±3.0	54.8±1.7
Mo75/PI 428215	8.3±2.5	129.2±4.9	14.5±1.0	36.0±3.0	2.1±0.2	16.7±1.2	33.3±2.3	32.7±2.5
Mo75/PI 428315	22.5±4.9	99.0±2.8	11.5±3.5	34.0±1.4	2.2±0.2	28.0±1.6	25.0±1.4	45.0±1.4

Table S4. Summarizes the main steps for developing and characterizing the synthetic durum–einkorn amphiploids.

Step	Description	Key Materials/Methods
1. Screening	*Bgt* race E09 inoculation at seedling stage	21 einkorn accessions; susceptible control Fielder
2. Hybridization	Cross between durum wheat (female) and resistant einkorn (male)	LDN, Mo75 (durum); 7 resistant einkorn accessions
3. Chromosome doubling	Colchicine treatment of F_1 seedlings	0.1% colchicine with 1–2% DMSO, 4–5 h
4. Resistance evaluation (seedling)	Inoculation with *Bgt* race E09 in growth chamber	Susceptible control Fielder; IT scale 0–4
5. Resistance evaluation (adult)	Field trial with natural *Bgt* pressure	Susceptible spreader rows inoculated with Bgt E09
6. Cytogenetic analysis	Chromosome counting and GISH	Probe: einkorn DNA; Blocking: LDN DNA
7. Marker analysis	PCR with functional/linked markers for *Pm* genes	Primer for *Pm1a*, *Pm3b*, *Pm4d*, *Pm37*, *pmNCA6*, *Pm60*,
8. Agronomic evaluation	Field trial measuring morphological traits	15 seeds per line; traits: height, tillering, spike length, TKW

References

16. Schmolke, M., Mohler, V., Hartl, L., Zeller, F. J., Hsam, S. L. A new powdery mildew resistance allele at the Pm4 wheat locus transferred from einkorn (*Triticum monococcum*). *Mol. Breed.* **2012**, 29(2), 449-456. https://doi.org/10.1007/s11032-011-9561-2

17. Jin, Y., Li, W., Li, Y., Li, D., Yan, H., Chen, S., Ma, P. Pm37 as a susceptible Sr22 allele confers resistance to wheat powdery mildew and leaf rust. *Nat. Commun.* **2026**, 17:3165. https://doi.org/10.1038/s41467-026-69717-1

18. Wan, W., Zhao, R., Zhao, P., Tang, Q., Lv, G., Chen, T., Bie, T. MutExomeSeq accelerates the cloning of PmNCA6 conferring powdery mildew resistance from *Triticum boeoticum*. *Engineering* **2026**, in press. https://doi.org/10.1016/j.eng.2026.02.027

49. Hewitt, T., Müller, M. C., Molnár, I., Mascher, M., Holušová, K., Šimková, H., Zhang, P. A highly differentiated region of wheat chromosome 7AL encodes a *Pm1a* immune receptor that recognizes its corresponding AvrPm1a effector from *Blumeria graminis*. *New Phytol.* **2021**, 229(5), 2812-2826. https://doi.org/10.1111/nph.17075

50. Tommasini, L., Yahiaoui, N., Srichumpa, P., Keller, B. Development of functional markers specific for seven Pm3 resistance alleles and their validation in the bread wheat gene pool.

Theor. Appl. Genet. **2026**, 114(1), 165-175. https://doi.org/10.1007/s00122-006-0420-1

51. Zhao, F., Li, Y., Yang, B., Yuan, H., Jin, C., Zhou, L., Shen, Q. H. Powdery mildew disease resistance and marker-assisted screening at the Pm60 locus in wild diploid wheat *Triticum urartu*. *Crop J.* **2020**, 8(2), 252-259. https://doi.org/10.1016/j.cj.2019.09.007
